# Supplementary material for: Disease-related and age-related changes of anterior chamber angle structures in patients with primary congenital glaucoma: An in vivo high-frequency ultrasound biomicroscopy-based study
Source: PLoS One. 2020 Jan 28;15(1):e0227602. doi: 10.1371/journal.pone.0227602 (PMC6986727; doi:10.1371/journal.pone.0227602)
Supplement: Attachment — Submitted filename: Response to reviewers.docx [file pone.0227602.s004.docx]

Please note the following minor comments (and the Reviewer 2 comments below) and please address all points:

1. Please confirm that the iris thickness measurements were taken perpendicular to the posterior iris plane and include this information in the Methods of the manuscript.

The iris thickness measurements were taken perpendicular to the posterior iris plane, we have included this information in the Methods of the manuscript. (Lines 127-128)

2. Please indicate if trabecular iris space area (TISA) measurements (mm2) were included during the study - these are usually reported for UBM studies (either at 500um or 700um from the scleral spur). If the TISA measurements were taken, please include in the manuscript and discuss the outcomes.

In patients with PCG, the dysgenesis of trabecular meshwork mainly affects the angle recess, which makes angle measurement involving the labeling of scleral spur difficult. So TISA measurement may not be accurate. On the other hand, TIA better illustrates the dysgenesis of trabecular meshwork and has been broadly used for patients with PCG. (Kobayashi H, Ono H, Kiryu J, Kobayashi K, Kondo T. Ultrasound biomicroscopic measurement of development of anterior chamber angle. Br J Ophthalmol. 1999;83(5):559-562)

3. Line 54: please amend to 'physiological.

This has been corrected. (Line 50)

4. Line 95: 'For those with unilateral disease, the unaffected contralateral eyes served as the control group (Group 2).'

We have corrected this sentence. (Line 89-90)

5. Line 175: 'Interobserver agreement was calculated by comparing initial values of Observer 1 (YS)

to those of Observer 2 (CX).'

We have corrected this sentence. (Lines 163-164)

5. Line 382: 'Analysis of the intraobserver and interobserver reproducibility was high.'

We have corrected this sentence. (Line 355)

7. Line 390: 'Fourthly,...'

This has been corrected. (Line 363)

Reviewers' comments:

Reviewer #2: I thank the authors for their comprehensive response. I have a few minor comments below.

Line 127: I presume that the iris thickness was measured perpendicularly to the retroiridal iris plane?

The iris thickness measurements were taken perpendicular to the posterior iris plane, we have included this information in the Methods of the manuscript. (Lines 127-128)

My point about trabecular-iris angle was related more to why trabecular-iris space area was not used to account for iris surface anatomy. TISA is commonly used and I wonder why it has been omitted here.

See above.

Line 359: spelling error

This has been corrected. (Line 363)

With regard to intraocular pressure, how do the authors explain the big difference in pressure between Groups 1 and 2 even though there was no relationship found cross sectional area of Schlemm's canal (Figure 3)? Could it be related to the degree to which the canals were closed/narrow?

We agree with the reviewer that the IOP may affect the cross-sectional area of Schlemm’s canal (SC) as previous study revealed in POAG (Mu L, Yin Z, Yan X, Hong Z. The Relationship between the 24-hour Fluctuations in Schlemm’s Canal and Intraocular Pressure: An Observational Study using High-Frequency Ultrasound Biomicroscopy. Curr Eye Res. 2017;42(10):1389-1395.) But in this study, IOP was not related with the size of the largest-measured SC area in pediatric patients with PCG. One explanation is that patients with PCG have maldeveloped SC, which could be segmental. The degree of the maldeveloped SC may lead to elevated IOP, which we don’t have enough data in this study to prove this. We only performed UBM at 4 o’clock, not 360 degree. The future development of circumference SC image would help solve this question. We have discussed it in the Discussion section. (Lines 296 to 303)
